# Appropriate use criteria for cardiovascular magnetic resonance imaging (CMR): SIC—SIRM position paper part 1 (ischemic and congenital heart diseases, cardio-oncology, cardiac masses and heart transplant)

**DOI:** 10.1007/s11547-020-01332-6

**Published:** 2021-02-24

**Authors:** Gianluca Pontone, Ernesto Di Cesare, Silvia Castelletti, Francesco De Cobelli, Manuel De Lazzari, Antonio Esposito, Marta Focardi, Paolo Di Renzi, Ciro Indolfi, Chiara Lanzillo, Luigi Lovato, Viviana Maestrini, Giuseppe Mercuro, Luigi Natale, Cesare Mantini, Aldo Polizzi, Mark Rabbat, Francesco Secchi, Aurelio Secinaro, Giovanni Donato Aquaro, Andrea Barison, Marco Francone

**Affiliations:** 1 Centro Cardiologico Monzino IRCCS, Milan, Italy; 2 Department of Life, Healt and Enviromental Sciences, University of L’Aquila, L’Aquila, Italy; 3 Center for the Cardiac Arrhythmias of Genetic Origin, Istituto Auxologico Italiano IRCCS, Milan, Italy; 4 School of Medicine, Vita-Salute San Raffaele University, Milan, Italy; 5 Department of Radiology, IRCCS San Raffaele Scientific Institute, Milan, Italy; 6 Department of Cardio-Thoraco-Vascular Sciences and Public Health, University of Padua, Padua, Italy; 7 Department of Cardiology, Azienda Ospedaliera Universitaria Senese, Siena, Italy; 8 U.O.C. Radiologia, Ospedale “San Giovanni Calibita” Fatebenefratelli - Isola Tiberina, Rome, Italy; 9 Division of Cardiology, University Magna Graecia, Italy and Mediterranea Cardiocentro, Naples, Italy; 10 Cardiologia Policlinico Casilino, Rome, Italy; 11 Cardiovascular Radiology Unit, Department of Imaging S.Orsola, Malpighi University Hospital, Bologna, Italy; 12 Department of Clinical Internal, Anesthesiologic and Cardiovascular Sciences, Sapienza University of Rome, Rome, Italy; 13 Department of Medical Sciences and Public Health, University of Cagliari, Cagliari, Italy; 14 Department of Diagnostic Imaging, Oncological Radiotherapy, and Hematology – Diagnostic Imaging Area, Rome, Italy; 15 Fondazione Policlinico Universitario Agostino Gemelli IRCCS, Rome, Italy; 16 Universita ` Cattolica del Sacro Cuore, Rome, Italy; 17 Department of Neuroscience, Imaging and Clinical Sciences, “G. d’Annunzio” University, Chieti, Italy; 18 Unit of Radiodiagnostics II, University Hospital “Policlinico-Vittorio Emanuele”, Catania, Italy; 19 Loyola University of Chicago, Chicago, USA; 20 Edward Hines Jr. VA Hospital, Hines, IL USA; 21 Department of Biomedical Sciences for Health, Università degli Studi di Milano, Milan, Italy; 22 Unit of Radiology, IRCCS Policlinico San Donato, San Donato Milanese, Italy; 23 Advanced Cardiovascular Imaging Unit, Department of Imaging, Bambino Gesù Children’s Hospital, Rome, Italy; 24 Fondazione Toscana G. Monasterio, Pisa, Italy; 25 Department of Radiological, Oncological and Pathological Sciences, Sapienza University of Rome, Rome, Italy; 26 Department of Biomedical Sciences, Humanitas University, Via Rita Levi Montalcini 4, 20090 Milan, Pieve Emanuele Italy

**Keywords:** Cardiac magnetic resonance, Appropriate use criteria, Consensus document, Cardiology, Radiology, Congenital heart disease, Ischemic heart disease, Cardio-oncology and toxic cardiomyopathy, Cardiac masses, Cardiac transplant

## Abstract

Cardiac magnetic resonance (CMR) has emerged as new mainstream technique for the evaluation of patients with cardiac diseases, providing unique information to support clinical decision-making. This document has been developed by a joined group of experts of the Italian Society of Cardiology and Italian society of Radiology and aims to produce an updated consensus statement about the current state of technology and clinical applications of CMR. The writing committee consisted of members and experts of both societies who worked jointly to develop a more integrated approach in the field of cardiac radiology. Part 1 of the document will cover ischemic heart disease, congenital heart disease, cardio-oncology, cardiac masses and heart transplant.

## Introduction

Since its initial utilization during early 1980s, cardiovascular magnetic resonance imaging (CMR) has evolved from a niche modality into a new mainstream tool, changing diagnostic paradigms in various cardiovascular settings. It provides unique information to support clinical decision-making, allows accurate prognostic stratification and has proven to be highly cost-effective in different scenarios [1]. Both radiological and cardiological skills are pivotal in CMR process to reach a patient-centered approach. For this reason, several international CMR training programs exist in both radiology and cardiology communities to improve the competence and to certify the skills [2–5]. Many documents supporting the utilization of CMR need to be updated frequently [6–8]. Thus, the present article is structured in the form of a consensus document which blends the competencies of a group of selected experts from the Italian Society of Cardiology (SIC) and Italian Society of Radiology (SIRM). The aim of the initiative is to produce a uniform and updated document, which would serve as guidance to our national healthcare community, with the goal of promoting a more efficient allocation of health care resources for CMR imaging in Italy.

### Definition of appropriateness and applied methodology

Articles are structured to define CMR appropriateness for the first diagnosis and follow-up in various clinical scenarios.

First, the writing committee discussed the table of content and assigned referrals for each chapter. Second, each referral conducted literature searches and drafted the assigned section, highlighting indications and rating them according to the following scores:

- Strong recommendation: there is evidence, general agreement, or both, that the test is useful (benefit ≫ risk).
- Moderate recommendation: there is conflicting evidence or opinion about the usefulness of the test; the weight of evidence/opinion however, is strongly in favor of the test’s usefulness. (benefit > risk).
- Weak recommendation: the test’s usefulness is less well established; there is a small net benefit (Benefit ≥ risk).
- No recommendation: there is evidence or general agreement that the risk/harm outweighs benefits (Benefit = or < risk).
- Expert opinion: there is insufficient evidence or evidence is unclear or conflicting, but this is what the working group recommends. Further research is recommended in this area.

As the third step, assigned scores were agreed in consensus by all authors and unanimously approved.

## Ischemic heart disease

Two scenarios including patients symptomatic for stable chest pain and patients presenting as acute coronary syndrome (ACS) such as ST elevation myocardial infarction (STEMI) will be considered. The main clinical indications for ischemic heart disease (IHD) are summarized in Table 1.

**Table 1:** Clinical recommendations for ischemic heart disease

Clinical setting	Diagnostic step	Recommendation	Report key-points
Stable chest pain in patients without history of revascularization	1st diagnosis	C	Detection of origin and proximal course of coronary arteries Evaluation of stenosis of proximal segments of coronary arteries
Stable chest pain in patients without history of revascularization	1st diagnosis	A	Detection of perfusion defects Detection of wall motion abnormalities Scar imaging Prognostic stratification
Stable chest pain in patients with previous history of revascularization and/or previous myocardial infarction	Follow-up	A	Detection of perfusion defects Detection of wall motion abnormalities Scar imaging for viability Prognostic stratification
Screening in asymptomatic patients with previous history of revascularization	Follow-up	C ( CMR recommended 3 years after PCI and 5 years after CABG)	Detection of perfusion defects Detection of wall motion abnormalities Scar imaging Detection of viability in case of previous myocardial infarction
Acute myocardial Infarction	1st diagnosis	B (CMR following revascularization)	Evaluation of left and right ventricle function Evaluation of area at risk and myocardial hemorrhage Evaluation of microvascular obstruction and necrotic area Post-infarction complications Prognostic stratification
Acute myocardial infarction	Follow-up	C	Evaluation of left and right ventricle function Evaluation of scar extent Post-infarction complications Prognostic stratification

*CABG* coronary artery bypass graft, *CMR* cardiac magnetic resonance, *PCI* percutaneous coronary intervention

### Stable chest pain

**Fig. 1:**
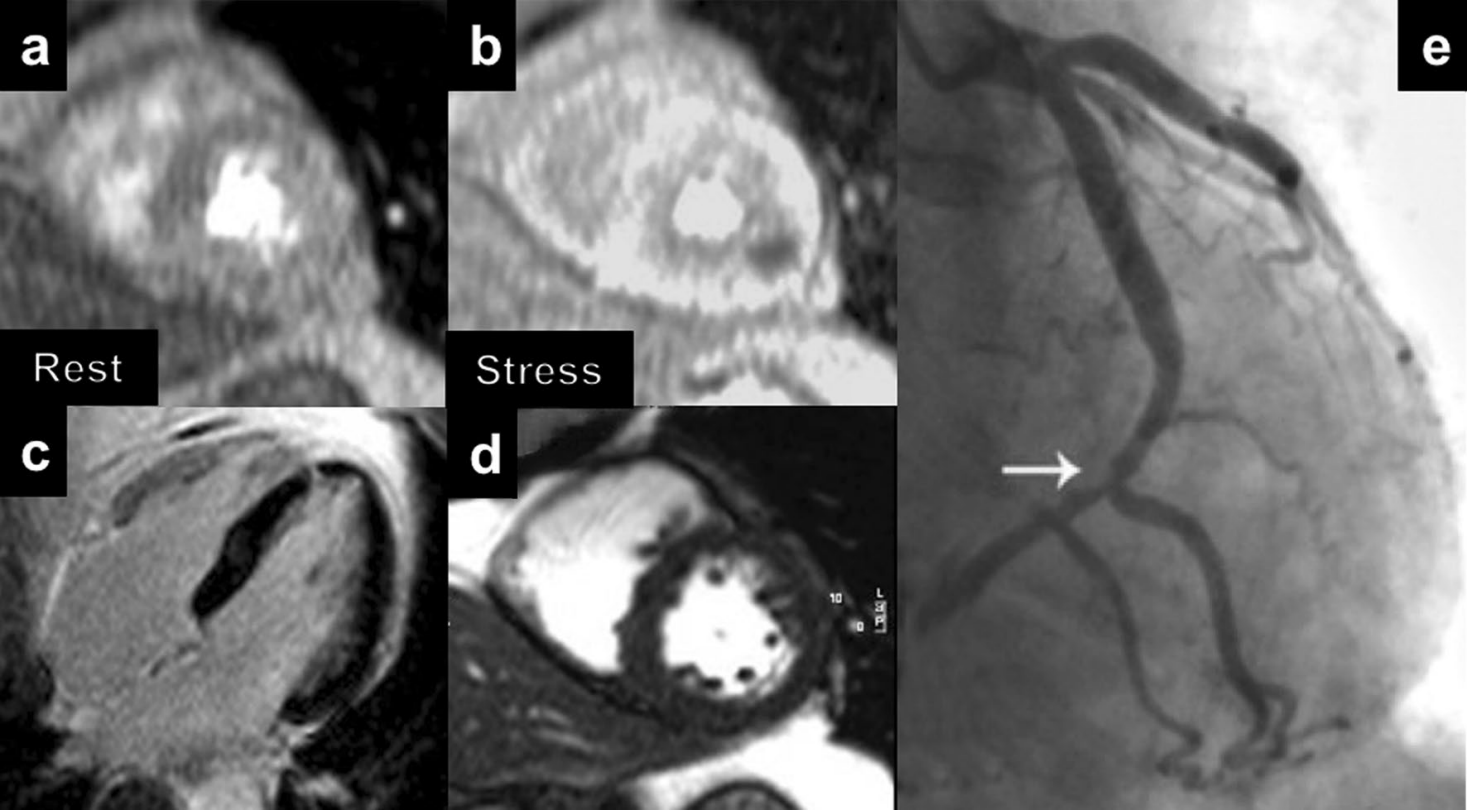
Example of stress CMR in a 54-year-old man with excertional chest pain. Rest (**a**) and stress (**b**) perfusion sequences show a large and reversible perfusion defect in the lateral mid LV wall. **c** No LGE is observed. **d** Biventricular global function was normal at cine-SSFP. **e** Coronary angiography confirmed a high grade stenosis of distal circumflex artery. CMR: cardiac magnetic resonance; LV: left ventricle; LGE: late gadolinium enhancement; SSFP: steady ste free-precession

CMR could be used to provide coronary artery imaging and reversible ischemia. The coronary artery imaging by CMR is achievable with non-contrast whole-heart coronary magnetic resonance angiography (MRA) that can provide visualization of the coronary tree within a single three-dimensional acquisition with an average sensitivity, specificity and negative predictive value of 88%, 72% and 88%, in a patient-based analysis, respectively [9]. However, coronary computed tomography angiography (CCTA) has emerged as a superior technique in this setting and the role of CMR should be limited only in the presence of a clear contraindication to CCTA. Regarding the detection of reversible ischemia, the main advantage of CMR is the possibility to evaluate perfusion defects, wall motion abnormalities and viability without the use of ionizing radiation (Fig. 1). In this setting, CMR can be considered appropriate for diagnostic and prognostic purposes [10, 11]. Regarding prognostic stratification, the evidence of reversible perfusion defects on stress perfusion is the strongest independent predictor for cardiovascular events [12, 13]. Finally, stress CMR is appropriate in stable chest pain in patients with a previous history of revascularization and more cost-effectiveness as compared to an anatomical strategy [14].

### STEMI

**Fig. 2:**
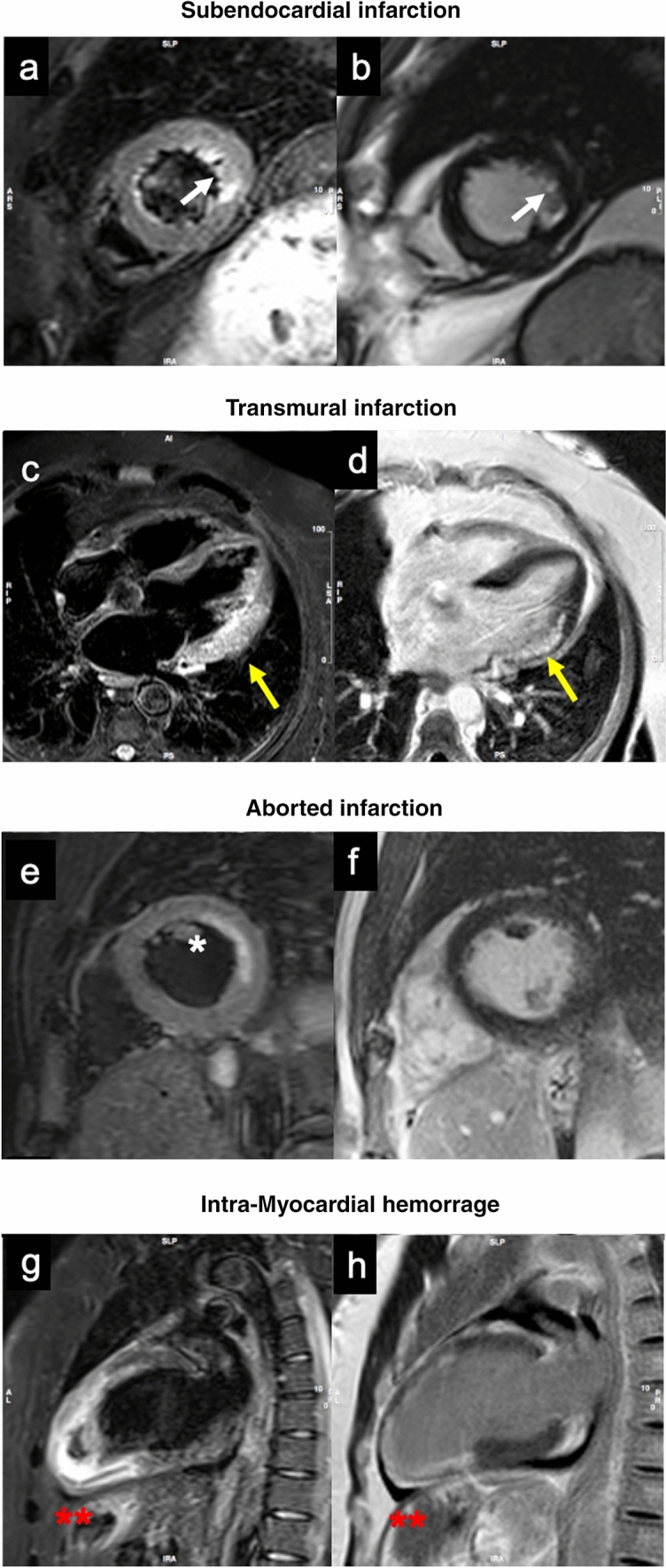
Examples of CMR patterns in STEMI and NSTEMI cases. A 52-years-old man with lateral NSTEMI **a**, **b** STIR and PSIR sequences show subendocardial edema (**a**) and LGE (**b**) involving the lateral wall at mid-apical level  (white arrows). **c**, **d** Patient with STEMI involving infero-lateral segments on basal and mid-ventricular plane. Transmural edema (**c**) and LGE (**d**) are present, with associated LV dilation and positive wall remodeling (yellow arrows). Aborted MI characterized by subendocardial mid-lateral  edema on short axis STIR image (**e**)  (*) , with no enhancement on LGE sequences (**f**). **g**, **h** 65-years-old man with critical stenosis of ADA and RCA. Anterior STEMI with anterior, septal and inferior wall involvement. IMH is visible in the inferior segments of apical plane (red **). NSTEMI: Non ST-elevation myocardial infarction; STIR: short tau inversion recovery; PSIR: phase sensitive inversion recovery; LGE: late gadolinium enhancement; STEMI: ST-elevation myocardial infarction; LV: left ventricle; MI: myocardial infarction; MVO: microvascular obstruction; ADA: anterior descending artery; RCA: right coronary artery; IMH: intramyocardial hemorrhage

In this setting, CMR can be helpful in both the diagnostic pathway and in prognostic stratification [15]. CMR is appropriate to detect the area at risk (AAR) that is defined as an ischemic territory that can be irreversibly damaged, if not reperfused, and can be easily depicted using T2 weighted black blood images [16, 17]. In addition, it can be used to detect intramyocardial hemorrhage (IMH) [13], which appears as signal loss within the area of myocardial infarction due to degradation products of hemoglobin. IMH is crucial for risk stratification with studies noting IMH as the most robust predictor of adverse left ventricular remodelling [18, 19]. Despite T2 weighted black blood images being widely used in clinical practice for the assessment of AAR, they showed some limitations such as artefacts related to slow blood flow, proximity of surface coil, high dynamic pattern of edema in the early stage of myocardial infarction and high image noise. In order to overcome these limitations, T1 and T2 mapping sequence have been developed and used in patients with acute myocardial infarction [20]. CMR is also appropriate in STEMI patients to detect the presence of microvascular obstruction (MVO) and the necrotic area by late gadolinium enhancement (LGE) imaging technique that is pivotal for prognostic stratification as well [21]. Finally, CMR has excellent diagnostic performance in detecting possible mechanical complications in patients with STEMI, although the use of CMR in this setting may be limited by the frequent unstable hemodynamic conditions of these patients. Figures 1 and 2 show explicative examples of CMR potentials in IHD patients.

## Congenital heart disease

### General indications

CMR can be appropriately used in the assessment of cardiac anatomy and function, blood flow, and extra-cardiac vascular structures in patients with simple and complex congenital heart disease (CHD). CMR is recommended for studying pediatric or juvenile populations that often require repeated follow-up examinations over time and is preferred in this context rather than computed tomography (CT) or cardiac catheterization, to avoid the use of iodinated contrast medium and ionizing radiation. Unfortunately, routine clinical use of CMR in new-borns and small children is hampered by the need of anesthesia. Despite echocardiography is the first line test in this setting, its image quality could be limited in several cases. Consequently, also considering its elevated reproducibility in the measurement of cardiac functional parameters, CMR has become a first-line investigation for several indications, especially in follow-up of surgical patients and in the evaluation of complex anomalies [22]. The main clinical indications for CHD are summarized in Table 2.

**Table 2:** Clinical recommendations for congenital heart disease

Clinical setting	Diagnostic step	Recommendation	Report key-points
Children < 6 years with CHD	1st diagnosis	B	CMR under general anesthesia, when other diagnostics are not conclusive
Children > 6 years with CHD	1st diagnosis	A	CMR superior to echocardiography
Urgent or critical patient	1st diagnosis	C	CMR and anaesthesiologic safety issues
	Follow-up	C	CMR and anaesthesiologic safety issues
Adult with CHD	1st diagnosis	A	CMR superior to echocardiography
	Follow-up	A	CMR superior to Echocardiography
Fetal CMR in CHD	1st diagnosis	N	Lack of standard CMR tools (ECG gating), protocols and sequences
Situs and systemic veins anomalies	1st diagnosis	A	CMR superior to echocardiography 3D sequences are accurate defining anatomy of pulmonary and systemic venous connection
	Follow-up	A	Cine SSFP and 3D sequences accurate defining systemic venous connections after Atrial Switch Operations (Mustard) or Fontan procedure
Cardiovascular shunt	1st diagnosis	A	PC flows are accurate for shunt quantification
Atrial septal defect	1st diagnosis	C	Echocardiography superior to CMR in detecting ASD I, II. 3D sequences are accurate defining anatomy of pulmonary veins
Atrial septal defect sinus venosus type	1st diagnosis	A	CMR is the diagnostic procedure with higher sensibility for Sinus Venosus subtype atrial defect
	Follow-up	A	Cine SSFP and 3D sequences are accurate defining anatomy of sinus venosus ASD. CMR is the Reference Standard for RV volumes and function
Anomalous pulmonary Venous connection	1st diagnosis	A	Cine SSFP and 3D sequences accurate defining pulmonary veins anatomy. CMR is gold standard for RV volumes and function
	Follow-up		Cine SSFP and 3D sequences are accurate defining pulmonary veins after repair. PC flows are accurate detecting abnormal flow distribution to lungs
Atrio-ventricular valve anomalies	1st diagnosis	C	Echocardiography is superior to CMR
Ebsteins’ anomaly	1st diagnosis	B	CMR is gold standard for RV volumes and function
Isolated VSD	1st diagnosis	C	PC flows are accurate for shunt quantification
Complex VSD	1st diagnosis	A	Delineate 3D anatomy
Ventricular diverticulum or aneurysm	1st diagnosis	A	CMR is the reference standard for myocardial tissue characterization
Tetralogy of fallot	1st diagnosis	D	Echocardiography is superior to CMR
	Follow up	A	PC flows are accurate for PV regurgitation quantification. CMR reference standard for RV function and volumes
Mediastinal pulmonary artery anomalies	1st diagnosis	A	3D sequences are accurate detecting abnormal pulmonary artery caliber and course (LPA sling)
	Follow-up		PC flows are accurate detecting abnormal flow distribution to lungs
Peripheral pulmonary arteries stenosis	1st diagnosis	B	CTA is superior to CMR CMR is less accurate evaluating lung parenchyma
Systemic to pulmonary collateral vessels	1st diagnosis	A	3D sequences are accurate for anatomy of pulmonary collaterals. PC flows are accurate for shunt quantification
Transposition of great arteries	1st diagnosis	D	Critical/unstable neonates. Echocardiography is generally superior to CMR
	Follow-up	A	3D sequence and cine SSFP are accurate assessing outflow tracts anomalies Stress CMR is accurate for ischemia detection after arterial switch operation
Aortic arch anomalies	1st diagnosis	A	Equivalent to CTA, limited in the evaluation of vessel/airways relationships
Aortic coarctation	1st diagnosis	A	Cine SSFP and 3D sequences are accurate defining anatomy. PC flows are accurate detecting obstructive flow profile (diastolic tail) at descending aorta. Aortic Valve and intracranial vessel evaluation is also recommended
	Follow-up	A	3D sequences are accurate for anatomy. CTA is superior to CMR for stent assessment
PDA associated with complex CHD	1st diagnosis	A	3D sequences are accurate detecting PDA anatomy PC flows are accurate for shunt quantification
Coronary anomalies screening in children and adolescents	1st diagnosis	A	CMR equivalent to CCTA, but radiation free and there is no need for contrast. CCTA is superior to CMR depicting course of coronaries (anatomical relationships, intramurality)
Coronary, ischemia and viability assessment	Follow-up	A	CMR is gold standard for myocardial tissue characterization. Stress CMR is accurate for ischemia detection after surgical correction of coronary anomalies

*ASD* atrial septal defect, *CHD* congenital heart disease, *CMR* cardiac magnetic resonance, *CTA* computed tomography angiography, *CCTA* coronary computed tomography angiography, *ECG* electrocardiogram, *LPA* left pulmonary artery, *PC* phase contrast, *PDA* patent ductus arteriosus, *RV* right ventricle, *SSFP* steady state free procession sequence, *VSD* ventricular septal defect

### Clinical indications in CHD

#### Anomalies of situs and systemic veins

CMR performs better than echocardiography in the analysis of visceral situs (solitus, inversus, ambiguous) and anatomy. The multi-planar nature and wide field of view of CMR enables a good appreciation of the whole thoracic and abdominal structures in a few images. CMR is particularly accurate in identifying malformations and assessing the systemic venous return during preoperative evaluation [23].

#### Atrial anomalies and anomalies of pulmonary veins

CMR is reliable in the diagnosis and overall assessment of atrial septal defects (ASD), although transoesophageal echocardiography (TOE) represents the gold standard in this setting. CMR also correlates with cardiac catheterization for the invasive quantification of shunts. CMR overcomes the limitations of the other imaging modalities in the presence of atypical defects, like sinus venosus or when associated with a partial anomalous pulmonary venous return (PAPVR) [24]. Accordingly, CMR is indicated for patients with isolated right ventricular dilatation to exclude a PAPVR or an ASD. Conversely, CMR is inferior to both TOE and trans-thoracic echocardiography (TTE) in the evaluation of patent foramen ovale (PFO) [25].

#### Atrio-ventricular connections, atrio-ventricular valves and ventricles anomalies

CMR is accurate in the study of discordant atrio-ventricular connections, valve atresia or atrio-ventricular defects and ventricular septal defects (VSD). However, it does not add significant data to echocardiography except for shunt quantification. CMR is useful in the presence of VSD associated with complex anomalies and in the preoperative evaluation of complex CHD. CMR may provide additional information in Ebstein’s disease (associated lesions, ventricular fibrosis) and may be indicated in the follow-up of right ventricular structure and function [26].

#### Valve anomalies

CMR is able to accurately quantify valvular regurgitation and stenosis by using phase contrast (PC) imaging sequences with adequate correlation to other traditional imaging modalities and it is highly reproducible. For this reason, CMR plays a central role in the serial follow-up of pulmonary regurgitation for corrected Tetralogy of Fallot (TOF) patients as well as for valvular or surgical conduit stenosis [27].

#### Anomalies of the great vessels

CMR is an extremely accurate method for studying all the diseases of the aorta, allowing precise measurements of aortic dimensions and the extent and morphology of the disease. MRA is well suitable for studying aortic arch anomalies, particularly utilizing 3D electrocardiogram (ECG) -gating with respiratory navigator sequences [23]. Location and severity of narrowing are accurately determined by CMR in aortic coarctation both through the morphologic visualization by 3D MRA and by calculating the flow velocities and gradients using PC sequences at the site of coarctation. Furthermore, CMR precisely quantifies the flow in the collateral circulation and even depicts the obstructive flow profile. CMR identifies complications of corrective surgery, such as pseudoaneurysms at the site of the surgical patch, and it is of greater value when a patent ductus arteriosus (PDA) is associated with complex anomalies in adults. CMR is accurate in the study of pulmonary arteries and their main branches which is an essential part of the preoperative and postoperative assessments of numerous CHD, such as TOF, pulmonary atresia and univentricular heart. MRA is the main technique for their evaluation, measuring dimensions, identifying stenosis, and for precise definition of the systemic-pulmonary collaterals, outcomes of procedures, and systemic-pulmonary shunts [23, 28].

#### Postoperative evaluation of CHD

This is the most well-established indication of CMR given its high reproducibility in studying biventricular function and considering that serial functional evaluation is crucial in the follow-up of these patients. Right ventricular function evaluation is of outmost importance in the follow-up of surgically managed CHD, such as TOF, pulmonary atresia with VSD, and transposition of the great arteries (TGA) treated with atrial switch. Other information exclusively provided by CMR is the depiction of myocardial fibrosis by LGE imaging, which stratifies risk of arrhythmias for these patients [29]. CMR is particularly important in the follow-up of TOF and associated anomalies. In patients with an atrial switch procedure (e.g., Senning, Mustard operation) for TGA, CMR is important in evaluating the morphology of the venous baffles and quantifying stenosis or shunts. Furthermore, CMR is the most accurate method in studying the function of the systemic right ventricle and the degree of myocardial fibrosis in order to stratify prognosis [30]. In patients with arterial switch procedure, CMR can accurately detect complications such as pulmonary artery compression or coronary artery re-implantation related problems. In CHD with a univentricular correction (e.g., hypoplastic left heart syndrome) CMR is ideal to delineate the complex morphology of cavopulmonary anastomosis (Fontan procedure) using contrast-enhanced 3D MRA or 3D steady-state-free precession (SSFP) imaging [26]. CMR allows the depiction of complications such as conduit thrombosis or stenosis. In particular, CMR flow sequences enable the quantification of flow patterns through the anastomosis and flow distribution to the right and left lung, and they are helpful in quantifying shunts in cases of systemic-pulmonary collaterals.

#### Coronary anomalies

Coronary MRA allows an accurate depiction of the origin and proximal course of the coronary arteries without the administration of contrast medium and ionizing radiation [31, 32].

**Fig. 3:**
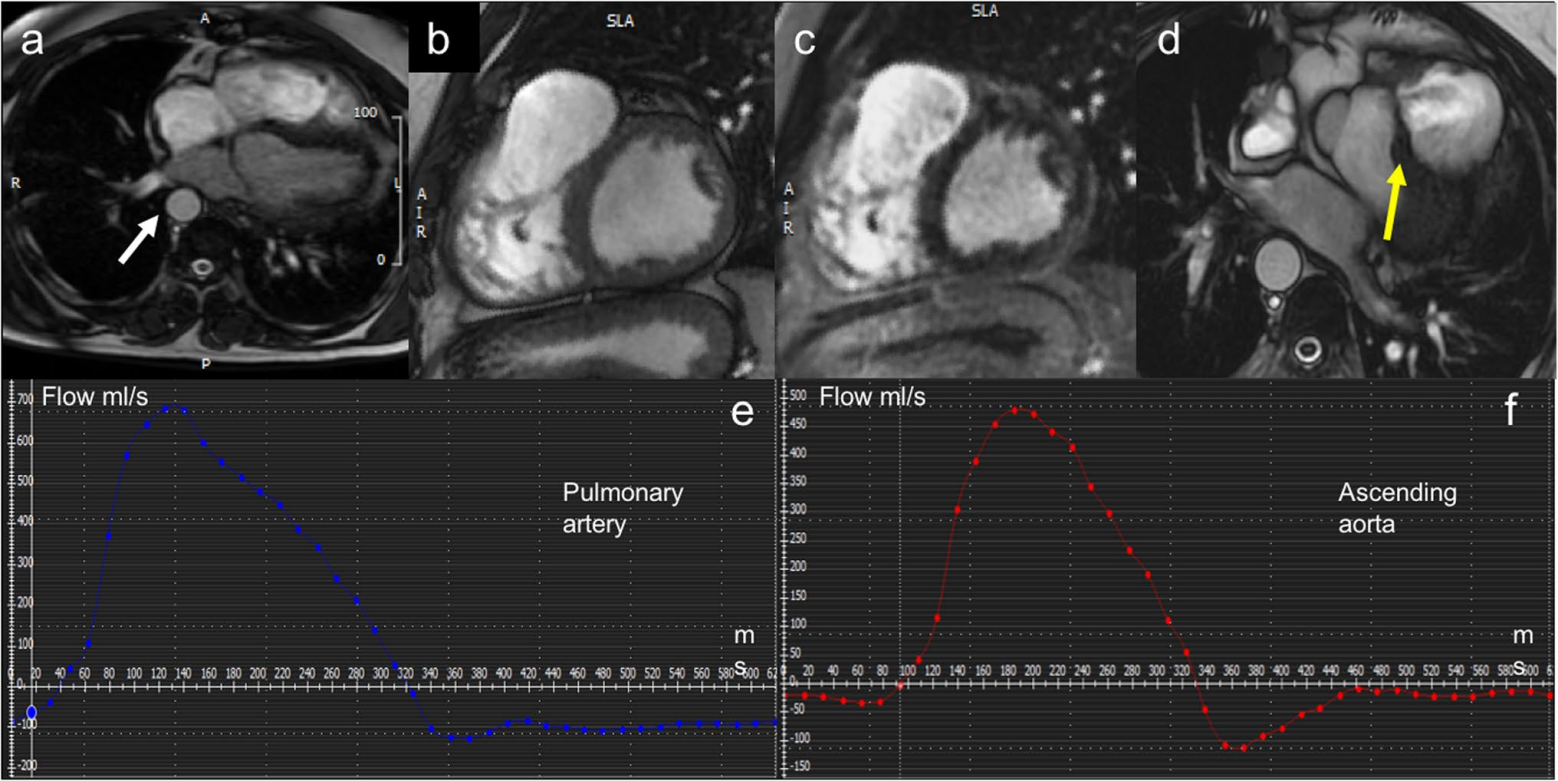
Example of CHD: CMR quantification of extra-cardiac shunt. **a** CMR Axial Heart SSFP images show dextroposition of aorta (white arrow). **b** Cine LV Sax images show right ventricular outflow tract patch. **c** Sax MDE sequences show a small amount of LGE around right ventricular outflow. The patient had ventricular arrhythmias with LBBB morphology. **d** Axial Heart SSFP images show residual VSD (yellow arrow). PC CMR of pulmonary artery (**e**) and ascending aorta (**f**) show Qp/Qs (1,49) meaning moderate shunt; moderate pulmonary regurgitation (RF 27%). CHD: congenital heart disease; CMR: cardiac magnetic resonance; SSFP: steady-state free precession; LGE: late gadolinium enhancement; LV: left ventricle; SAX: short axis; PC: phase contrast; VSD: ventricular septal defect; RF: regurgitant fraction; LBBB: left bundle branch block

Figure 3 shows an  example of CMR quantification of an extra-cardiac shunt.

## Cardio-oncology and toxic cardiomyopathy

Advances in cancer treatment led to improved survival of patients but have also increased morbidity and mortality due to treatment side effects. Cardiotoxicity can be classified in type 1 (i.e., anthracycline) characterized by dose dependent myocardial injury, more likely to be irreversible, and type 2 (i.e., trastuzumab) with a higher likelihood of recovery after discontinuation of the offending agent. Anthracyclines are one of the most studied examples of cardiotoxicity as this class of drug has high efficacy for treatment of solid tumors and hematological malignancies but may also cause irreversible cardiac damage, which can be acute, early or late, affecting prognosis [33].

Cardiotoxicity is defined as a decline of left ventricle ejection fraction (LVEF) ≥ 5% from baseline in symptomatic patients or ≥ 10% in asymptomatic patients to less than 55%. The 2014 Expert Consensus Statement for Multimodality Imaging Evaluation of Adult Patients during and after cancer therapy from the American Society of Echocardiography and the European Association of Cardiovascular Imaging updated the definition of cancer therapeutics-related cardiac dysfunction as a decrease in LVEF of > 10% to a value < 53%.

CMR is recognized as a method to screen for chemotherapy-related cardiotoxicity in case of poor transthoracic echocardiography (TTE) image quality due to its accuracy, reproducibility and ability to detect subtle changes in right and left ventricular function [34]. CMR also evaluates myocardial edema through T2w imaging and T2 mapping, diffuse and focal myocardial fibrosis through T1 mapping/extracellular volume fraction (ECV) and LGE [34, 35].

Diffuse myocardial fibrosis induced by anthracycline therapy can occur several years after completion of treatment and it can be assessed by T1 mapping [36]. However, an early decrease of T1 value 48 h after first treatment with anthracyclines can predict the development of anthracycline cardiomyopathy after completion of chemotherapy [37].

Some small studies using CMR have also shown myocardial edema early following anthracycline therapy by using T2-weighted sequences. The presence of edema has been associated with persistent reduction in RV function in follow-up examinations [35].

Finally, ECV is also increased in patients after anthracyclines therapy as compared to healthy controls [38].

CMR is also appropriate in monitoring cardiac involvement in cancer-related treatment, providing distinct bio-signatures of early inflammatory involvement (raised native T1 and T2) and interstitial fibrosis and remodelling (raised native T1 but not T2), thus providing an algorithm allowing to identify susceptible myocardium to potentially guide cardio-protective treatment measures [39].

Moreover, radiation therapy improves cancer-related outcomes in a variety of malignancies such as lymphoma, breast, lung, and head and neck cancers. Radiation-induced heart disease is a serious side effect of cancer treatment, which may manifest as pericarditis (acute and subacute), pericardial effusions or late effects (10–15 years after exposure) related to cardiovascular fibrosis which can lead to diverse clinical manifestations including heart failure, constrictive pericarditis, restrictive cardiomyopathy, valvular abnormalities, premature coronary disease and arrhythmias [40]. The main clinical indications for cardio-oncology are summarized in Table 3.

**Table 3:** Clinical recommendations for cardio-oncology

Clinical setting	Diagnostic step	Recommendation	Report key-points
Cardiotoxicity	1st diagnosis	B	Typically used if poor TTE image quality prohibits measurement of LVEF or if LVEF is < 53% Detection of subclinical declines in right and left ventricular function (Cine imaging) Detection of diffuse (T1 mapping and ECV evaluation) or focal (LGE) myocardial fibrosis Detection of myocardial edema (T2w imaging and T2 mapping)
	Follow-up	B	Typically used if poor TTE image quality prohibits measurement of LVEF or if LVEF is < 53% Detection of subclinical declines in right and left ventricular function (Cine imaging) Detection of diffuse (T1 mapping and ECV evaluation) or focal (LGE) myocardial fibrosis Detection of myocardial edema (T2w imaging and T2 mapping)
Radiotoxicity	1st diagnosis	B	Detection of pericarditis (acute and subacute), constrictive pericarditis, restrictive cardiomyopathy Detection of subclinical declines in cardiac function (cine imaging) Detection of diffuse (T1 mapping and ECV evaluation) or focal (LGE) Detection of myocardial edema (T2w imaging and T2 mapping)
	Follow-up	B	Detection of pericarditis (acute and subacute), constrictive pericarditis, restrictive cardiomyopathy Detection of subclinical declines in cardiac function (cine imaging) Detection of diffuse (T1 mapping and ECV evaluation) or focal (LGE) Detection of myocardial edema (T2w imaging and T2 mapping)

*ECV* extracellular volume, *LGE* late gadolinium enhancement, *LVEF* left ventricle ejection fraction, *TTE* transthoracic echocardiography

## Cardiac masses

CMR offers the most comprehensive approach to cardiac masses as it allows to determine the location, pathological substrate, lesion mobility, dynamic perfusion and hemodynamic impact of a suspected cardiac tumor.

**Fig. 4:**
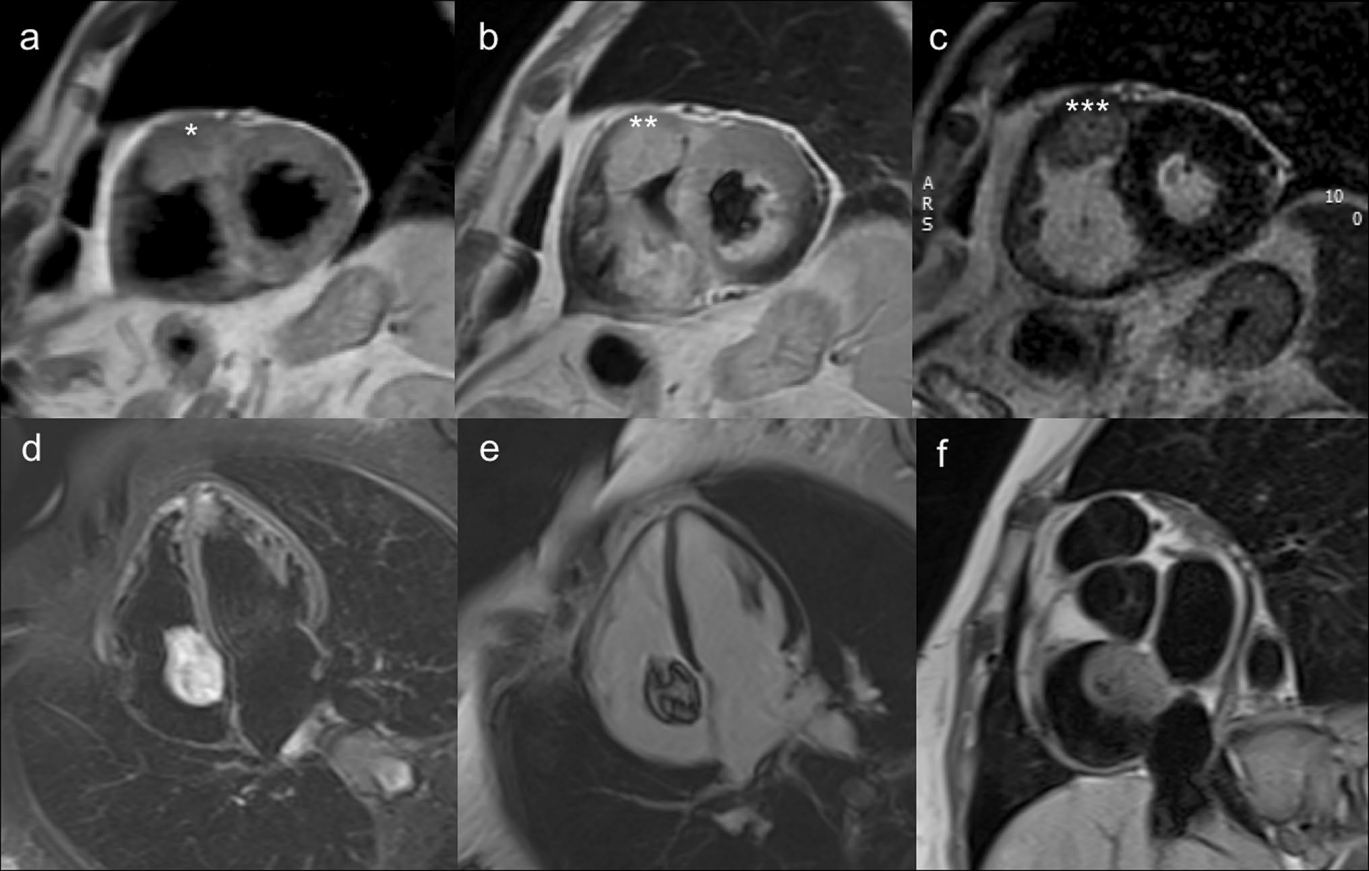
Examples of a typical malignant (angiosarcoma: **a**–**c**) and benign (myxoma: **d**–**f**) cardiac mass. **a**–**c** Angiosarcoma appears as a large, infiltrative lesion attached to the right atrial roof and extending in the pericardial space (***). There is inhomogeneous contrast enhancement in post-contrast T1-weighted and LGE images (**b**–**c**) as compared to pre-contrast T1-weighted short axis image  (***). Atrial myxoma on T1 weighted short axis image (**d**), shows spotty low signal intralesional components, consistent with presence of intratumoral calcifications (*). Lesion appears typically hyperintese in T2 weighted images (**e**), due to the myxoid tissue content of the mass. LGE sequences show inhomogeneous enhancement of the tumor  (**f**). LGE: Late Gadolinium Enhancement

CMR is recommended in the diagnostic process and its contribution in the clinical workup is summarized as follows:

- Differentiation between non-tumoral versus tumoral lesions: anatomic pitfalls refer to the presence of normal cardiac structures or embryological remnants, which can easily be misinterpreted as cardiac masses. These include, among others, the presence of prominent or hypertrophic structures like the moderator band, Eustachian valves, Chiari network, crista terminalis or false cordae tendine. Among pseudomasses (real masses of non-neoplastic origin or benign cardiac or extra-cardiac changes that mimic a disease), the list of differentials is long and covers a large and heterogeneous series of congenital and acquired conditions. Most common pseudo-tumors comprise intracavitary thrombi, areas of lipomatous hypertrophy, infective or coelomic cysts and abscesses and large hiatal hernias [41]. CMR can recognize these structures and support clinical-decision making.
- Differential diagnosis between benign and malignant lesions (Fig. 4): besides the presence of well-established imaging criteria (i.e., lesion size, infiltrative expansion, irregular margins, contrast enhancement) discrimination between benign versus malignant cardiac tumors remains challenging, often requiring biopsy correlation [42]. This is attributable to the heterogeneity of the underlying histological substrate of different masses, resulting in an often unpredictable signal behavior and post-contrast enhancement appearance. Besides the fact that certain CMR features such as intralesional perfusion and contrast enhancement may be predictive of a pathologic diagnosis, these characteristics may highly overlap between different types of highly vascular benign (e.g., haemangiomas or vascular anomalies) and malignant masses (e.g., hypervascular secondary malignancies, angiosarcoma or various malignant neuroendocrine tumors). Associated findings are often helpful in this regard, and may include the presence of pleural or pericardial effusions and extra-cardiac concomitant disease (e.g., pulmonary or skeletal masses) [42].
- Surgical planning: due to the high risk of distal embolization, sudden cardiac death (SCD), and hemodynamic collapse, surgical treatment is often indicated in cardiac tumors and require careful preoperative staging. Patients with primary malignant diseases or metastases may undergo surgery for symptomatic and palliative treatment (i.e., palliative mass debulking) whereas radical resection can often be obtained in benign masses. CMR is fundamental to evaluate tumor characteristics, for planning of the preferred surgical approach and reconstruction of the cardiac chambers [43].
- Detection and follow-up of tumor recurrences: incidence of local recurrences largely depends on tumor histology and adequacy of surgical excision, reaching a cumulative incidence of up to 13% for cardiac myxomas [44]. CMR superior tissue characterization capabilities can detect early disease recurrences and to monitor disease progression by accurately measuring volumetric mass changes.
- Detection and characterization of myocardial damage following oncological treatments (radiotherapy or chemotherapy; see dedicated paragraph).

CMR appropriateness is limited in the evaluation of small lesions attached to fast moving structures. This can be observed in the evaluation of valvular masses, like papillary fibro-elastomas or infective vegetations, which are barely visible if smaller than 10 mm, because of the intrinsically low temporal resolution of cine sequences, as compared to an echocardiographic approach. The main clinical indications for cardiac mass evaluation are summarized in Table 4 and Fig. 4 shows some examples of cardiac masses detected by CMR [45].

**Table 4:** Clinical Recommendations for cardiac masses

Clinical setting	Diagnostic step	Recommendation	Report key-points
Differential diagnosis between pseudomasses, non-neoplastic lesions and tumors	1st diagnosis	A	Consider common and less common anatomic pitfalls (i.e., crista terminalis, Eustachian valve, moderator band etc.) Determine the neoplastic vs. non-neoplastic nature of a finding: consider clinical information, signal features and mass location
Differential diagnosis between benign and malignant masses	1st diagnosis	B	Consider typical hallmarks of malignancy: lesion size, infiltrative expansion, irregular margins, contrast enhancement, multiple foci etc. Consider extra-cardiac, ancillary signs (i.e., pulmonary masses, evidence of extra-thoracic metastatic lesions)
	Follow-up	B	Rule-out postoperative recurrences CMR following palliative chemotherapy or radiation therapy
CMR tissue characterization	1st diagnosis	C	Consider CMR intrinsic limitations and limited specificity of signal abnormalities in defining underlying histological substrate Be aware of typical anatomic locations of different cardiac masses Diagnosis feasible in few exceptions, mostly fat containing neoplastic lesions
Characterization of small mobile lesions	1st diagnosis	D	Small moving structured (e.g., valvular masses), barely visible with CMR because of the intrinsically low temporal resolution of the method A dimensional cut-off of 10 mm is usually considered a minimum threshold for their depiction Consider differential diagnoses of valvular vegetations (i.e., infective and/or thrombotic masses)
Local staging and preoperative planning	1st diagnosis	A	Evaluate local extension to surrounding organs, vascular structures, cardiac chambers and pericardium Resectability depends on the invasion CMR useful to guide surgical reconstruction of cardiac chambers

*CMR* cardiac magnetic resonance

## Cardiac transplant

Heart transplantation is a life-saving therapy for individuals with end-stage heart failure, congenital heart diseases, restrictive cardiomyopathy and infectious cardiac diseases. Acute rejection and accelerated coronary artery disease, considered as chronic rejection, represent the most common clinical problems. Despite stringent selection criteria and significant advances in anti-rejection therapy, the mortality rate is still very high. As result of a non-uniform pathologic process and of a difficult histological interpretation, surveillance with endomyocardial biopsies can underdiagnose the rejection. In the early stages it may occur without major cardiac dysfunction, therefore echocardiographic measurements may lack sensitivity; diastolic measures have shown correlation with acute rejection, however without uniform consistency [46].

In this context, the tissue characterization ability of CMR has suggested its use as a non-invasive tool to diagnose acute rejection. Compared to endomyocardial biopsy, CMR has shown positive results [47]; however, its use is still limited.

The value of T2-weighted imaging has been evaluated both in animal and human studies with inconsistent results [47, 48]. However, the use of technologies inferior to current standards and the low contrast-to-noise nature of the sequence may have affected the results. In initial studies, no differences were found in patients with biopsy proven rejection compared to those without rejection [47] and did not correlate with transplant rejection [49]. However, a more recent study on 50 patients has shown more positive results [48].

Late gadolinium enhancement is not sensitive and has not been widely investigated. In a small study including patients with different grades of rejection, a higher relative myocardial signal intensity in the early phase post-contrast, a marker of inflammatory, hyperemia, was observed [48].

The new mapping techniques may have an emerging role in the diagnosis of cardiac transplant rejection [50]. Myocardial T1 and T2 are increased in the acute phase following transplantation. T1-mapping has been shown to decrease after successful treatment [51] and to display excellent negative predictive value for the non-invasive detection of rejection [50]. An increase of myocardial T2 has been shown to predict acute rejection [52] with high sensitivity and specificity [52, 53] and to normalize after treatment [52]. Also a combined approach of T2-mapping and ECV quantification has been shown to help in guiding biopsies [51], potentially decreasing their routine number.

Myocardial ischemia, a component of the rejection process and transplant arteriopathy, lacks clinical symptoms. Adenosine stress perfusion shows a reduction in patients with a prior history of rejection compared with those without [54].

The main clinical indications for CMR in post-cardiac transplantation are summarized in Table 5.

**Table 5:** Clinical recommendations post-cardiac transplantation

Clinical setting	Diagnostic step	Recommendation	Report key-points
Acute rejection	1st diagnosis	C	Detection of myocardial edema with T2 weighted images Detection of myocardial edema with T2 mapping Detection of interstitial fibrosis with T1 mapping
Chronic rejection	1st diagnosis	C	Detection of perfusion defects with stress CMR

*CMR* cardiac magnetic resonance
